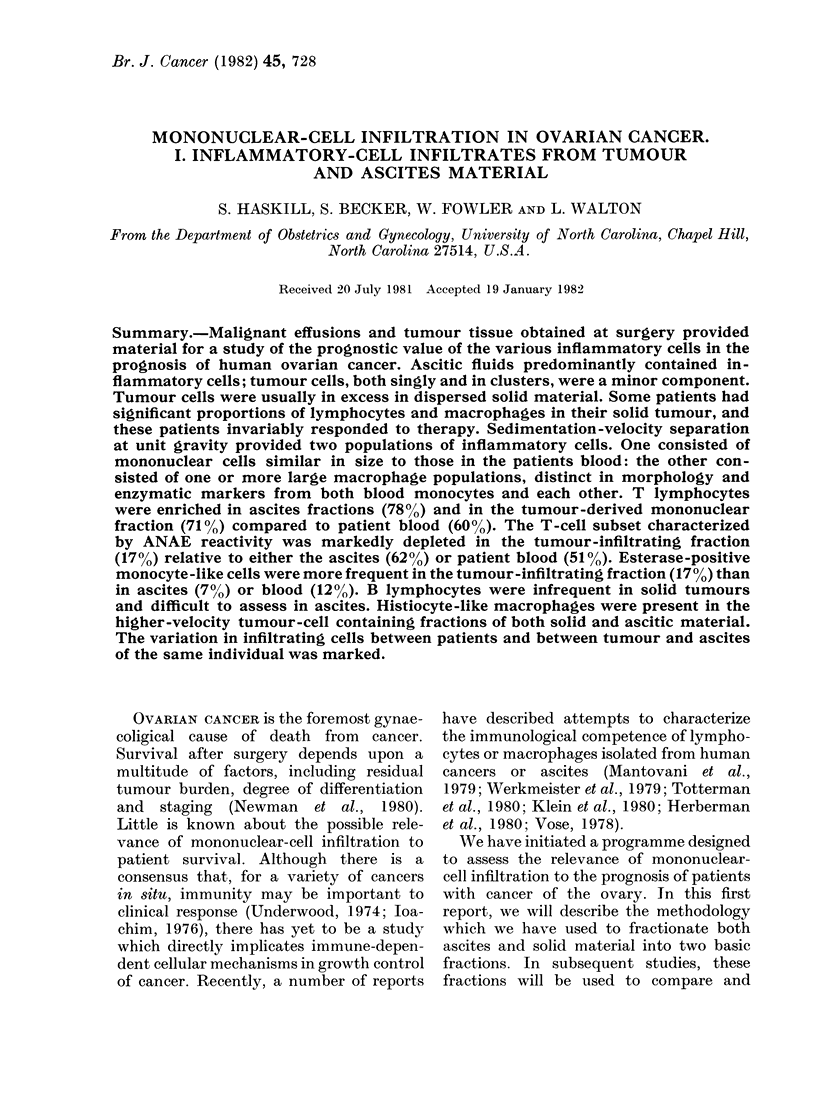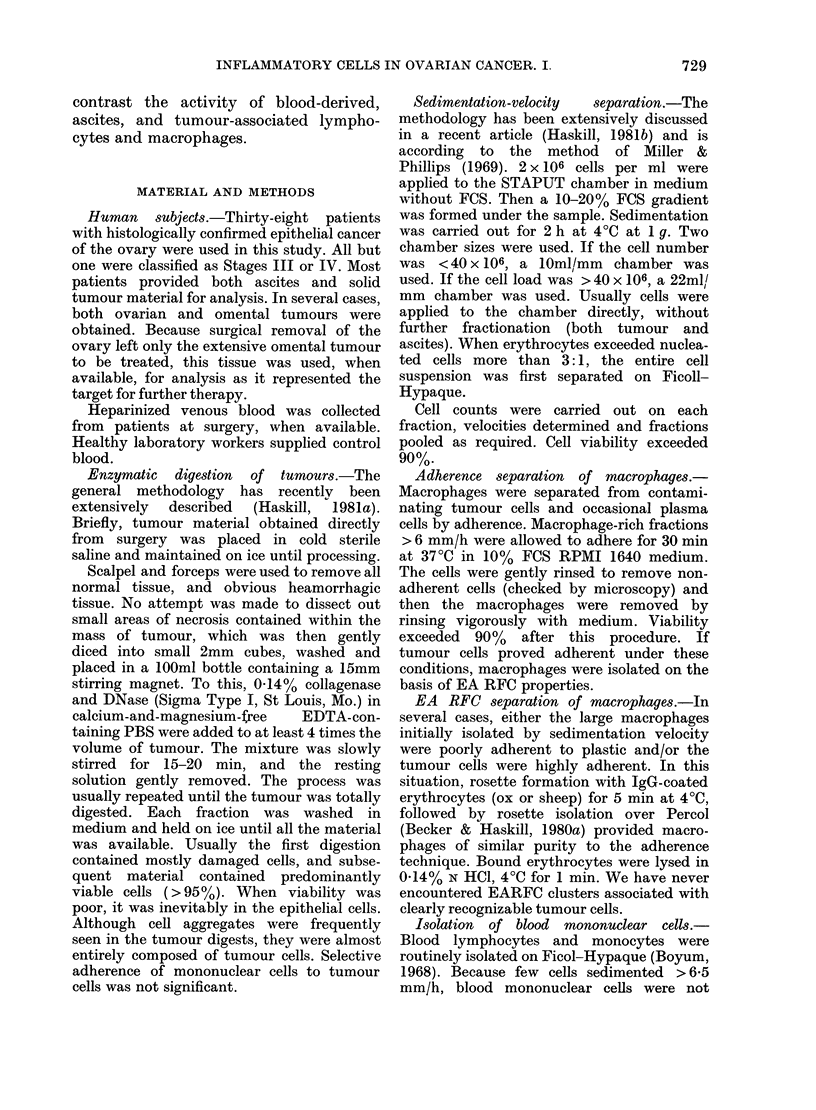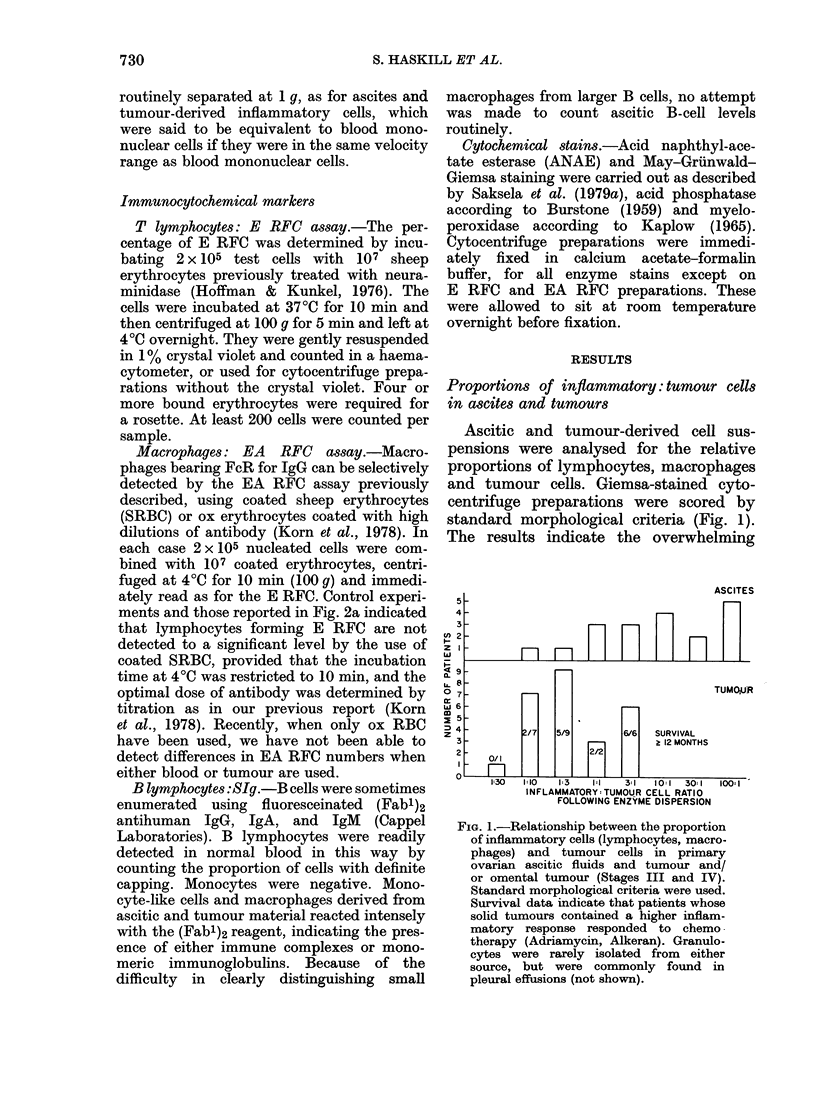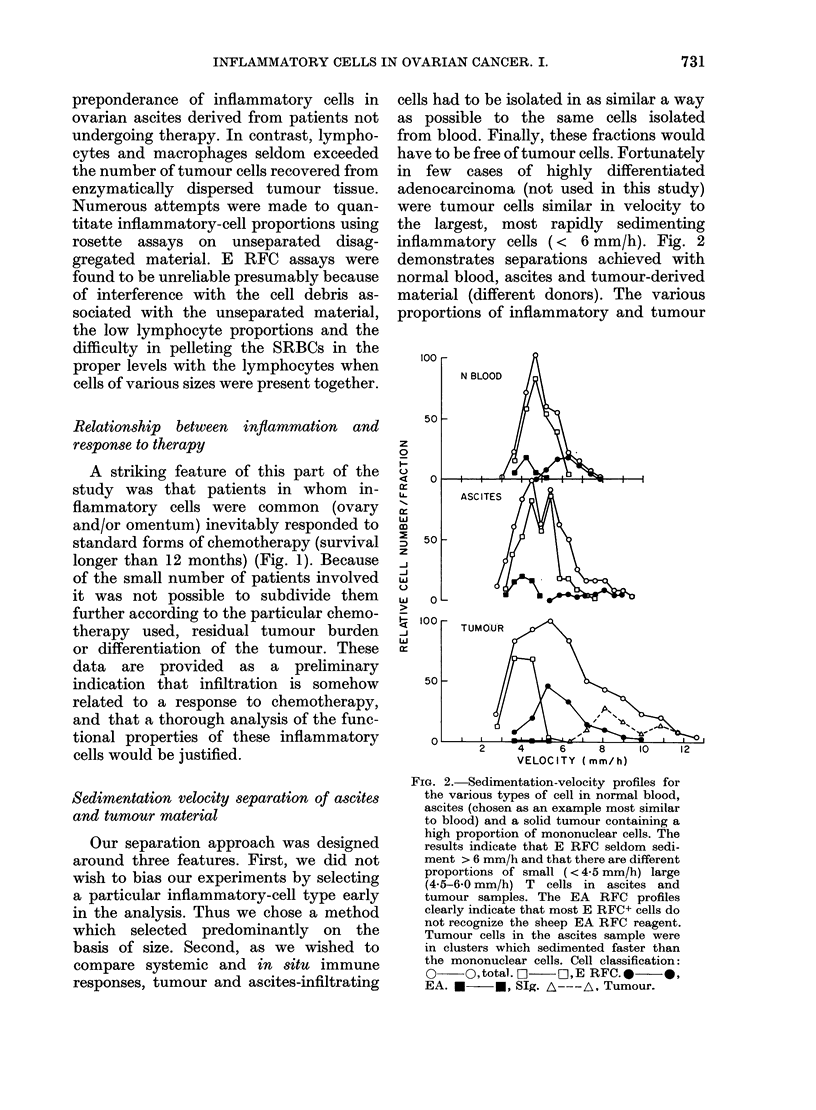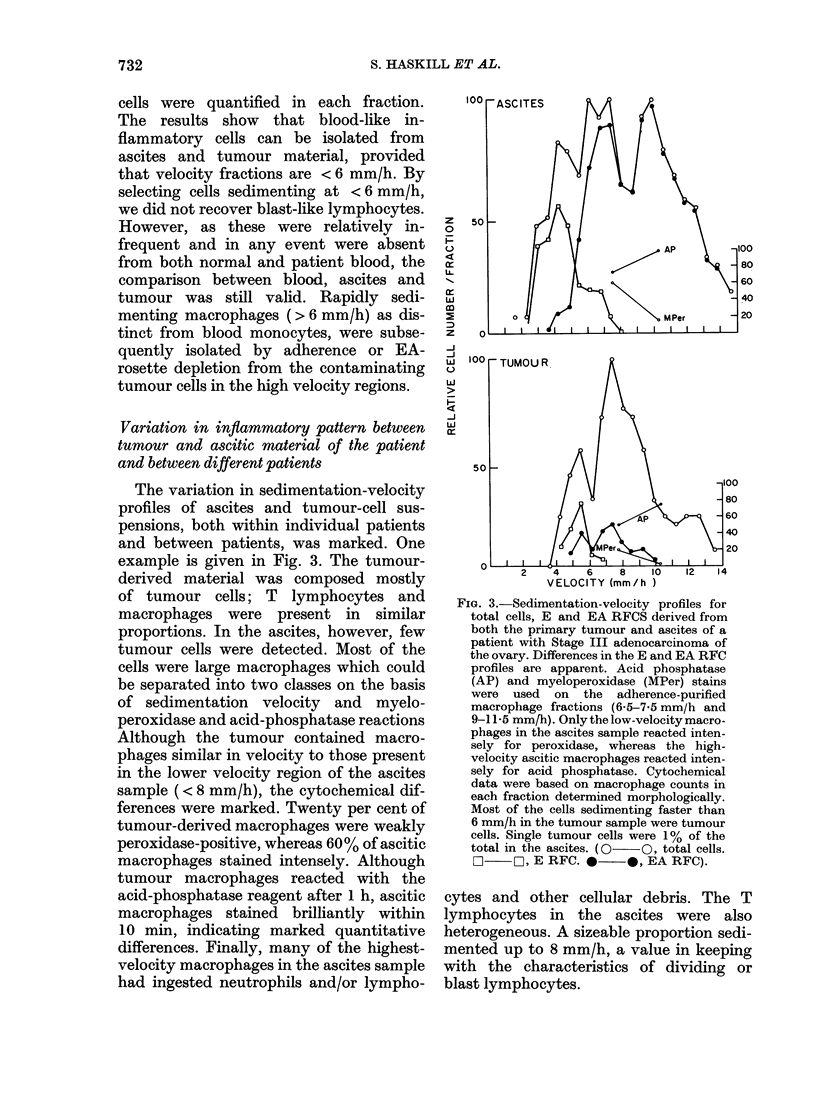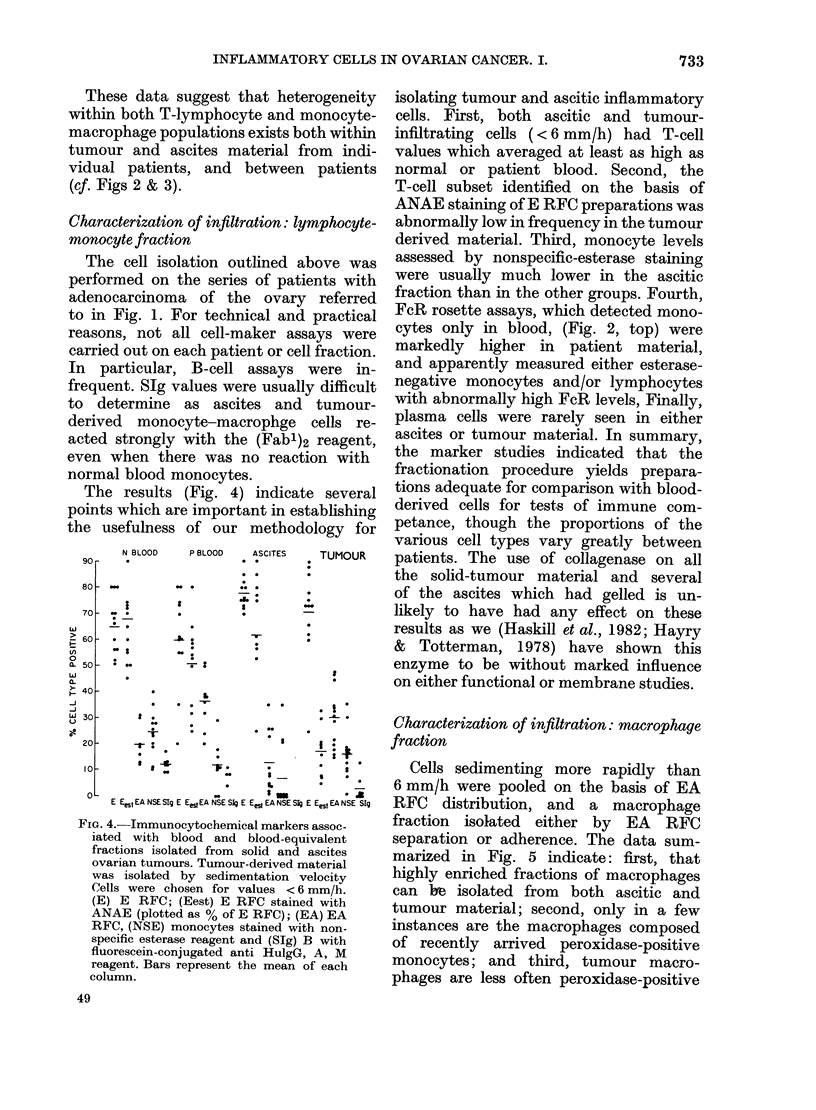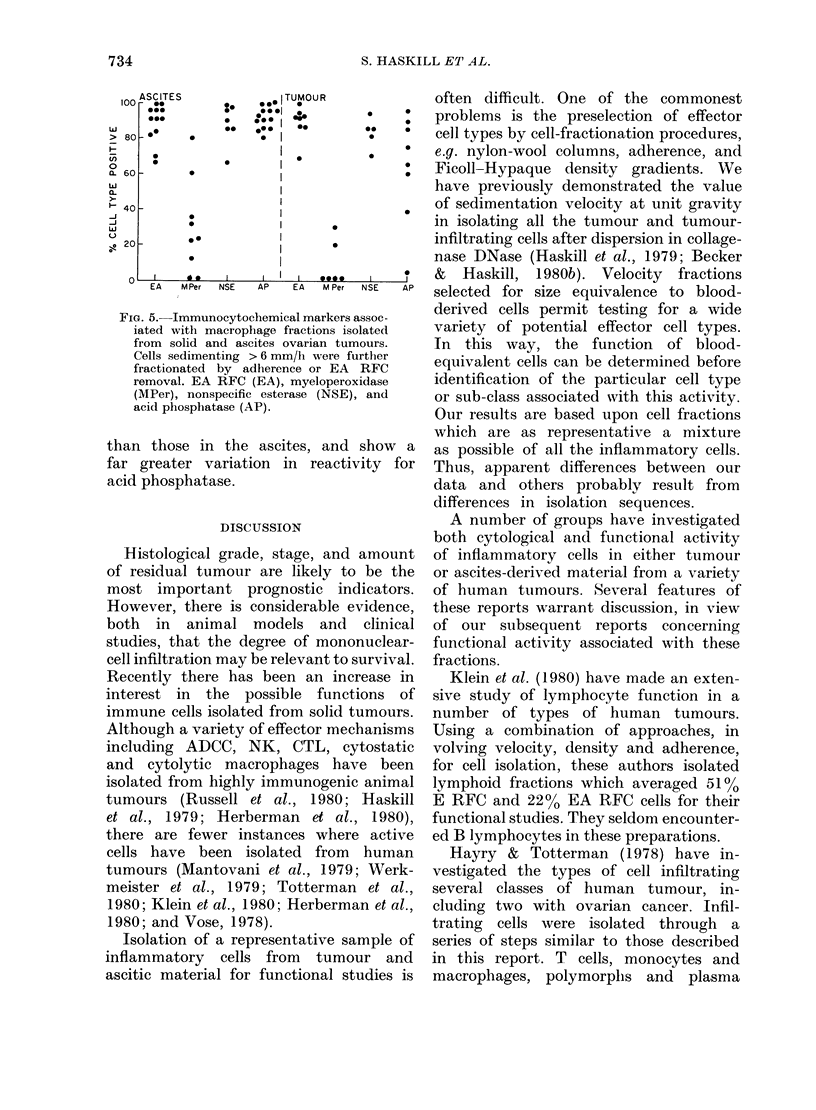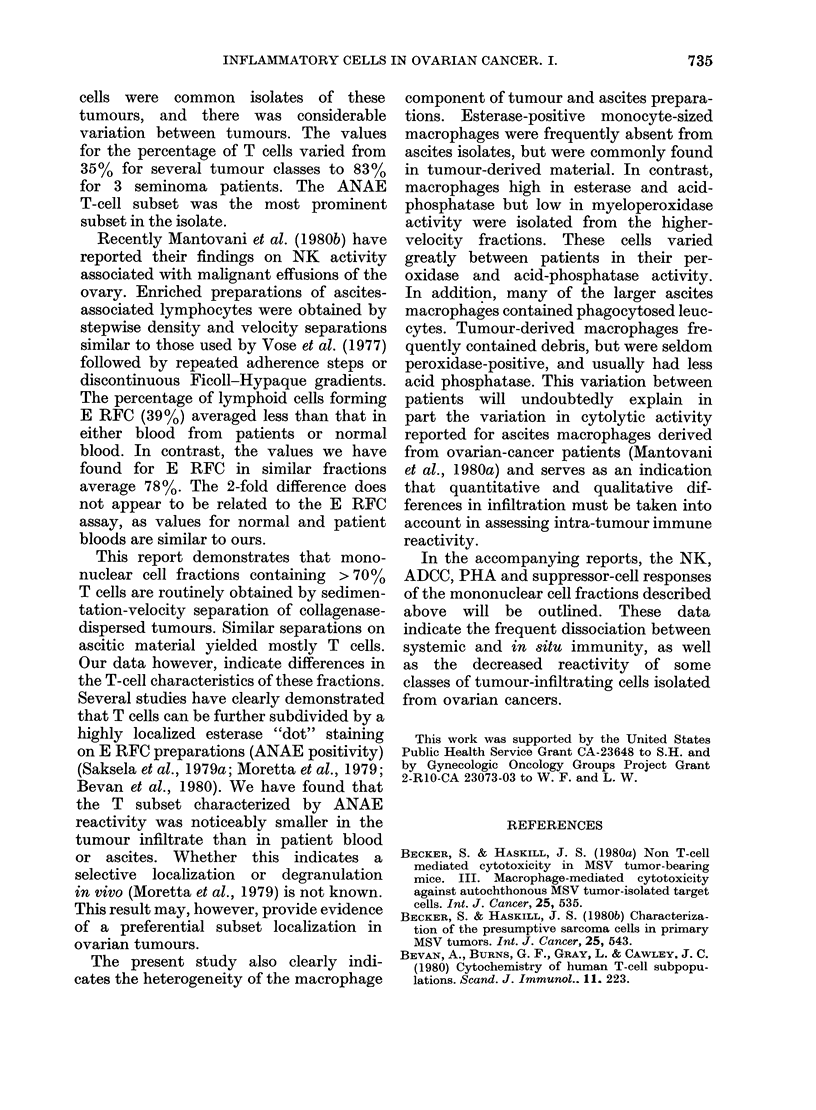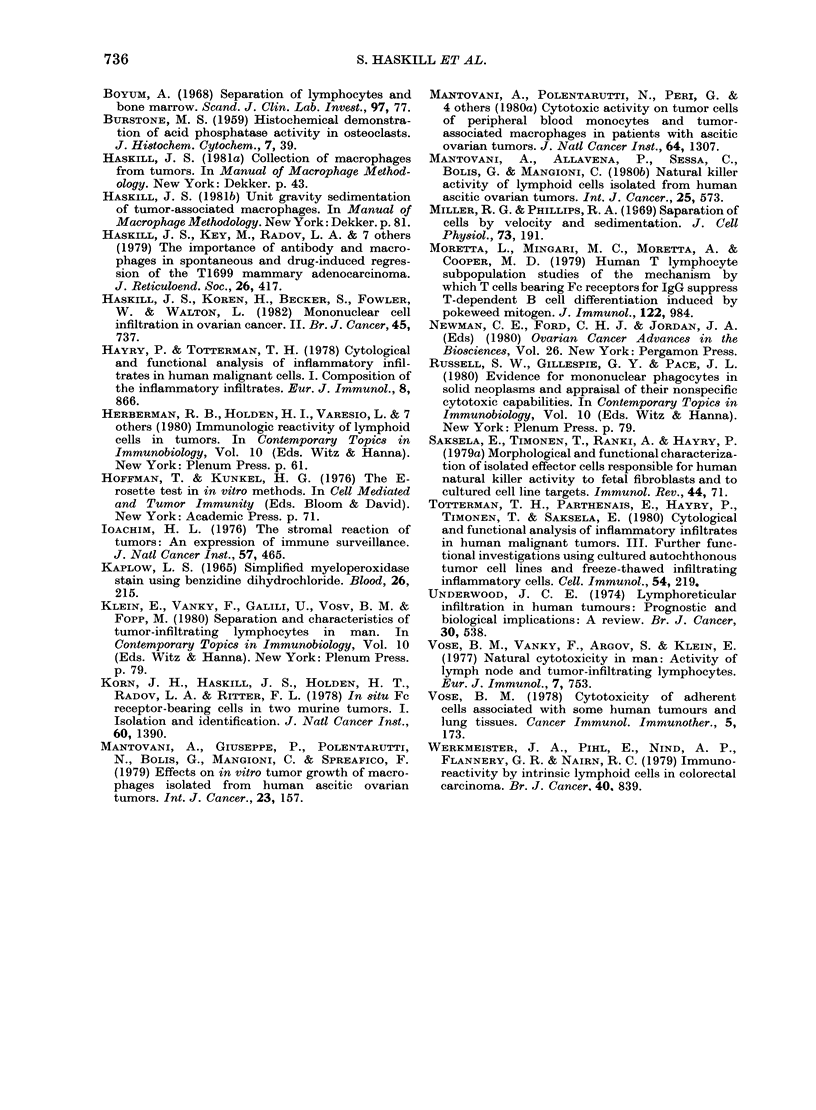# Mononuclear-cell infiltration in ovarian cancer. I. Inflammatory-cell infiltrates from tumour and ascites material.

**DOI:** 10.1038/bjc.1982.114

**Published:** 1982-05

**Authors:** S. Haskill, S. Becker, W. Fowler, L. Walton

## Abstract

Malignant effusions and tumour tissue obtained at surgery provided material for a study of the prognostic value of the various inflammatory cells in the prognosis of human ovarian cancer. Ascitic fluids predominantly contained inflammatory cells; tumour cells, both singly and in clusters, were a minor component. Tumour cells were usually in excess in dispersed solid material. Some patients had significant proportions of lymphocytes and macrophages in their solid tumour, and these patients invariably responded to therapy. Sedimentation-velocity separation at unit gravity provided tow populations of inflammatory cells. One consisted of mononuclear cells similar in size to those in the patients blood: the other consisted of one or more large macrophage populations, distinct in morphology and enzymatic markers from both blood monocytes and each other. T lymphocytes were enriched in ascites fractions (78%) and in the tumour-derived mononuclear fraction (71%) compared to patient blood (60%). The T-cell subset characterized by ANAE reactivity was markedly depleted in the tumour-infiltrating fraction (17%) compared to patient blood (62%) or patient blood (51%). Esterase-positive monocyte-like cells were more frequent in the tumour-infiltrating fraction (17%) than ascites (7%) or blood (12%). B lymphocytes were infrequent in solid tumours and difficult to assess in ascites. Histiocyte-like macrophages were present in the higher-velocity tumour-cell containing fractions of both solid and ascitic material. The variation in infiltrating cells between patients and between tumour and ascites of the same individual was marked.


*Br. J. Cancer* (1982) **45**, 728

### MONONUCLEAR-CELL INFILTRATION IN OVARIAN CANCER. I. INFLAMMATORY-CELL INFILTRATES FROM TUMOUR AND ASCITES MATERIAL

S. HASKILL, S. BECKER, W. FOWLER AND L. WALTON

*From the Department of Obstetrics and Gynecology, University of North Carolina, Chapel Hill, North Carolina* 27514, *U.S.A.*

Received 20 July 1981 Accepted 19 January 1982

**Summary.—Malignant effusions and tumour tissue obtained at surgery provided material for a study of the prognostic value of the various inflammatory cells in the prognosis of human ovarian cancer. Ascitic fluids predominantly contained inflammatory cells; tumour cells, both singly and in clusters, were a minor component. Tumour cells were usually in excess in dispersed solid material. Some patients had significant proportions of lymphocytes and macrophages in their solid tumour, and these patients invariably responded to therapy. Sedimentation-velocity separation at unit gravity provided two populations of inflammatory cells. One consisted of mononuclear cells similar in size to those in the patients blood: the other consisted of one or more large macrophage populations, distinct in morphology and enzymatic markers from both blood monocytes and each other. T lymphocytes were enriched in ascites fractions (78%) and in the tumour-derived mononuclear fraction (71%) compared to patient blood (60%). The T-cell subset characterized by ANAE reactivity was markedly depleted in the tumour-infiltrating fraction (17%) relative to either the ascites (62%) or patient blood (51%). Esterase-positive monocyte-like cells were more frequent in the tumour-infiltrating fraction (17%) than in ascites (7%) or blood (12%). B lymphocytes were infrequent in solid tumours and difficult to assess in ascites. Histiocyte-like macrophages were present in the higher-velocity tumour-cell containing fractions of both solid and ascitic material. The variation in infiltrating cells between patients and between tumour and ascites of the same individual was marked.**

Ovarian cancer is the foremost gynaecoligical cause of death from cancer. Survival after surgery depends upon a multitude of factors, including residual tumour burden, degree of differentiation and staging (Newman *et al.*, 1980). Little is known about the possible relevance of mononuclear-cell infiltration to patient survival. Although there is a consensus that, for a variety of cancers *in situ*, immunity may be important to clinical response (Underwood, 1974; Ioachim, 1976), there has yet to be a study which directly implicates immune-dependent cellular mechanisms in growth control of cancer. Recently, a number of reports have described attempts to characterize the immunological competence of lymphocytes or macrophages isolated from human cancers or ascites (Mantovani *et al.*, 1979; Werkmeister *et al.*, 1979; Totterman *et al.*, 1980; Klein *et al.*, 1980; Herberman *et al.*, 1980; Vose, 1978).

We have initiated a programme designed to assess the relevance of mononuclear-cell infiltration to the prognosis of patients with cancer of the ovary. In this first report, we will describe the methodology which we have used to fractionate both ascites and solid material into two basic fractions. In subsequent studies, these fractions will be used to compare and contrast the activity of blood-derived, ascites, and tumour-associated lymphocytes and macrophages.

### MATERIAL AND METHODS

*Human subjects.*—Thirty-eight patients with histologically confirmed epithelial cancer of the ovary were used in this study. All but one were classified as Stages III or IV. Most patients provided both ascites and solid tumour material for analysis. In several cases, both ovarian and omental tumours were obtained. Because surgical removal of the ovary left only the extensive omental tumour to be treated, this tissue was used, when available, for analysis as it represented the target for further therapy.

Heparinized venous blood was collected from patients at surgery, when available. Healthy laboratory workers supplied control blood.

*Enzymatic digestion of tumours.*—The general methodology has recently been extensively described (Haskill, 1981*a*). Briefly, tumour material obtained directly from surgery was placed in cold sterile saline and maintained on ice until processing.

Scalpel and forceps were used to remove all normal tissue, and obvious heamorrhagic tissue. No attempt was made to dissect out small areas of necrosis contained within the mass of tumour, which was then gently diced into small 2mm cubes, washed and placed in a 100ml bottle containing a 15mm stirring magnet. To this, 0·14% collagenase and DNase (Sigma Type I, St Louis, Mo.) in calcium-and-magnesium-free EDTA-containing PBS were added to at least 4 times the volume of tumour. The mixture was slowly stirred for 15–20 min, and the resting solution gently removed. The process was usually repeated until the tumour was totally digested. Each fraction was washed in medium and held on ice until all the material was available. Usually the first digestion contained mostly damaged cells, and subsequent material contained predominantly viable cells (>95%). When viability was poor, it was inevitably in the epithelial cells. Although cell aggregates were frequently seen in the tumour digests, they were almost entirely composed of tumour cells. Selective adherence of mononuclear cells to tumour cells was not significant.

*Sedimentation-velocity separation.*—The methodology has been extensively discussed in a recent article (Haskill, 1981*b*) and is according to the method of Miller & Phillips (1969). $2\times10^6$ cells per ml were applied to the STAPUT chamber in medium without FCS. Then a 10–20% FCS gradient was formed under the sample. Sedimentation was carried out for 2 h at 4°C at 1 *g*. Two chamber sizes were used. If the cell number was $<40\times10^6$, a 10ml/mm chamber was used. If the cell load was $>40\times10^6$, a 22ml/mm chamber was used. Usually cells were applied to the chamber directly, without further fractionation (both tumour and ascites). When erythrocytes exceeded nucleated cells more than 3:1, the entire cell suspension was first separated on Ficoll–Hypaque.

Cell counts were carried out on each fraction, velocities determined and fractions pooled as required. Cell viability exceeded 90%.

*Adherence separation of macrophages.*—Macrophages were separated from contaminating tumour cells and occasional plasma cells by adherence. Macrophage-rich fractions >6 mm/h were allowed to adhere for 30 min at 37°C in 10% FCS RPMI 1640 medium. The cells were gently rinsed to remove non-adherent cells (checked by microscopy) and then the macrophages were removed by rinsing vigorously with medium. Viability exceeded 90% after this procedure. If tumour cells proved adherent under these conditions, macrophages were isolated on the basis of EA RFC properties.

*EA RFC separation of macrophages.*—In several cases, either the large macrophages initially isolated by sedimentation velocity were poorly adherent to plastic and/or the tumour cells were highly adherent. In this situation, rosette formation with IgG-coated erythrocytes (ox or sheep) for 5 min at 4°C, followed by rosette isolation over Percol (Becker & Haskill, 1980*a*) provided macrophages of similar purity to the adherence technique. Bound erythrocytes were lysed in 0·14% N HCl, 4°C for 1 min. We have never encountered EARFC clusters associated with clearly recognizable tumour cells.

*Isolation of blood mononuclear cells.*—Blood lymphocytes and monocytes were routinely isolated on Ficol–Hypaque (Boyum, 1968). Because few cells sedimented >6·5 mm/h, blood mononuclear cells were not routinely separated at 1 *g*, as for ascites and tumour-derived inflammatory cells, which were said to be equivalent to blood mononuclear cells if they were in the same velocity range as blood mononuclear cells.

*Immunocytochemical markers*

*T lymphocytes: E RFC assay.*—The percentage of E RFC was determined by incubating $2 \times 10^5$ test cells with $10^7$ sheep erythrocytes previously treated with neuraminidase (Hoffman & Kunkel, 1976). The cells were incubated at 37 °C for 10 min and then centrifuged at 100 *g* for 5 min and left at 4 °C overnight. They were gently resuspended in 1% crystal violet and counted in a haemacytometer, or used for cytocentrifuge preparations without the crystal violet. Four or more bound erythrocytes were required for a rosette. At least 200 cells were counted per sample.

*Macrophages: EA RFC assay.*—Macrophages bearing FcR for IgG can be selectively detected by the EA RFC assay previously described, using coated sheep erythrocytes (SRBC) or ox erythrocytes coated with high dilutions of antibody (Korn *et al.*, 1978). In each case $2 \times 10^5$ nucleated cells were combined with $10^7$ coated erythrocytes, centrifuged at 4°C for 10 min (100 *g*) and immediately read as for the E RFC. Control experiments and those reported in Fig. 2a indicated that lymphocytes forming E RFC are not detected to a significant level by the use of coated SRBC, provided that the incubation time at 4°C was restricted to 10 min, and the optimal dose of antibody was determined by titration as in our previous report (Korn *et al.*, 1978). Recently, when only ox RBC have been used, we have not been able to detect differences in EA RFC numbers when either blood or tumour are used.

*B lymphocytes: SIg.*—B cells were sometimes enumerated using fluoresceinated $(Fab^1)_2$ antihuman IgG, IgA, and IgM (Cappel Laboratories). B lymphocytes were readily detected in normal blood in this way by counting the proportion of cells with definite capping. Monocytes were negative. Monocyte-like cells and macrophages derived from ascitic and tumour material reacted intensely with the $(Fab^1)_2$ reagent, indicating the presence of either immune complexes or monomeric immunoglobulins. Because of the difficulty in clearly distinguishing small macrophages from larger B cells, no attempt was made to count ascitic B-cell levels routinely.

*Cytochemical stains.*—Acid naphthyl-acetate esterase (ANAE) and May–Grünwald–Giemsa staining were carried out as described by Saksela *et al.* (1979*a*), acid phosphatase according to Burstone (1959) and myeloperoxidase according to Kaplow (1965). Cytocentrifuge preparations were immediately fixed in calcium acetate–formalin buffer, for all enzyme stains except on E RFC and EA RFC preparations. These were allowed to sit at room temperature overnight before fixation.

### RESULTS

*Proportions of inflammatory: tumour cells in ascites and tumours*

Ascitic and tumour-derived cell suspensions were analysed for the relative proportions of lymphocytes, macrophages and tumour cells. Giemsa-stained cytocentrifuge preparations were scored by standard morphological criteria (Fig. 1). The results indicate the overwhelming

FIG. 1.—Relationship between the proportion of inflammatory cells (lymphocytes, macrophages) and tumour cells in primary ovarian ascitic fluids and tumour and/or omental tumour (Stages III and IV). Standard morphological criteria were used. Survival data indicate that patients whose solid tumours contained a higher inflammatory response responded to chemotherapy (Adriamycin, Alkeran). Granulocytes were rarely isolated from either source, but were commonly found in pleural effusions (not shown).

preponderance of inflammatory cells in ovarian ascites derived from patients not undergoing therapy. In contrast, lymphocytes and macrophages seldom exceeded the number of tumour cells recovered from enzymatically dispersed tumour tissue. Numerous attempts were made to quantitate inflammatory-cell proportions using rosette assays on unseparated disaggregated material. E RFC assays were found to be unreliable presumably because of interference with the cell debris associated with the unseparated material, the low lymphocyte proportions and the difficulty in pelleting the SRBCs in the proper levels with the lymphocytes when cells of various sizes were present together.

### *Relationship between inflammation and response to therapy*

A striking feature of this part of the study was that patients in whom inflammatory cells were common (ovary and/or omentum) inevitably responded to standard forms of chemotherapy (survival longer than 12 months) (Fig. 1). Because of the small number of patients involved it was not possible to subdivide them further according to the particular chemotherapy used, residual tumour burden or differentiation of the tumour. These data are provided as a preliminary indication that infiltration is somehow related to a response to chemotherapy, and that a thorough analysis of the functional properties of these inflammatory cells would be justified.

### *Sedimentation velocity separation of ascites and tumour material*

Our separation approach was designed around three features. First, we did not wish to bias our experiments by selecting a particular inflammatory-cell type early in the analysis. Thus we chose a method which selected predominantly on the basis of size. Second, as we wished to compare systemic and *in situ* immune responses, tumour and ascites-infiltrating cells had to be isolated in as similar a way as possible to the same cells isolated from blood. Finally, these fractions would have to be free of tumour cells. Fortunately in few cases of highly differentiated adenocarcinoma (not used in this study) were tumour cells similar in velocity to the largest, most rapidly sedimenting inflammatory cells (< 6 mm/h). Fig. 2 demonstrates separations achieved with normal blood, ascites and tumour-derived material (different donors). The various proportions of inflammatory and tumour

FIG. 2.—Sedimentation-velocity profiles for the various types of cell in normal blood, ascites (chosen as an example most similar to blood) and a solid tumour containing a high proportion of mononuclear cells. The results indicate that E RFC seldom sediment > 6 mm/h and that there are different proportions of small (< 4·5 mm/h) large (4·5–6·0 mm/h) T cells in ascites and tumour samples. The EA RFC profiles clearly indicate that most E RFC$^+$ cells do not recognize the sheep EA RFC reagent. Tumour cells in the ascites sample were in clusters which sedimented faster than the mononuclear cells. Cell classification: ○——○, total. □——□, E RFC. ●——●, EA. ■——■, SIg. △---△, Tumour.

cells were quantified in each fraction. The results show that blood-like inflammatory cells can be isolated from ascites and tumour material, provided that velocity fractions are < 6 mm/h. By selecting cells sedimenting at < 6 mm/h, we did not recover blast-like lymphocytes. However, as these were relatively infrequent and in any event were absent from both normal and patient blood, the comparison between blood, ascites and tumour was still valid. Rapidly sedimenting macrophages ( > 6 mm/h) as distinct from blood monocytes, were subsequently isolated by adherence or EA-rosette depletion from the contaminating tumour cells in the high velocity regions.

### *Variation in inflammatory pattern between tumour and ascitic material of the patient and between different patients*

The variation in sedimentation-velocity profiles of ascites and tumour-cell suspensions, both within individual patients and between patients, was marked. One example is given in Fig. 3. The tumour-derived material was composed mostly of tumour cells; T lymphocytes and macrophages were present in similar proportions. In the ascites, however, few tumour cells were detected. Most of the cells were large macrophages which could be separated into two classes on the basis of sedimentation velocity and myeloperoxidase and acid-phosphatase reactions Although the tumour contained macrophages similar in velocity to those present in the lower velocity region of the ascites sample ( < 8 mm/h), the cytochemical differences were marked. Twenty per cent of tumour-derived macrophages were weakly peroxidase-positive, whereas 60% of ascitic macrophages stained intensely. Although tumour macrophages reacted with the acid-phosphatase reagent after 1 h, ascitic macrophages stained brilliantly within 10 min, indicating marked quantitative differences. Finally, many of the highest-velocity macrophages in the ascites sample had ingested neutrophils and/or lymphocytes and other cellular debris. The T lymphocytes in the ascites were also heterogeneous. A sizeable proportion sedimented up to 8 mm/h, a value in keeping with the characteristics of dividing or blast lymphocytes.

FIG. 3.—Sedimentation-velocity profiles for total cells, E and EA RFCS derived from both the primary tumour and ascites of a patient with Stage III adenocarcinoma of the ovary. Differences in the E and EA RFC profiles are apparent. Acid phosphatase (AP) and myeloperoxidase (MPer) stains were used on the adherence-purified macrophage fractions (6·5–7·5 mm/h and 9–11·5 mm/h). Only the low-velocity macrophages in the ascites sample reacted intensely for peroxidase, whereas the high-velocity ascitic macrophages reacted intensely for acid phosphatase. Cytochemical data were based on macrophage counts in each fraction determined morphologically. Most of the cells sedimenting faster than 6 mm/h in the tumour sample were tumour cells. Single tumour cells were 1% of the total in the ascites. (○——○, total cells. □——□, E RFC. ●——●, EA RFC).

These data suggest that heterogeneity within both T-lymphocyte and monocyte-macrophage populations exists both within tumour and ascites material from individual patients, and between patients (*cf.* Figs 2 & 3).

*Characterization of infiltration: lymphocyte-monocyte fraction*

The cell isolation outlined above was performed on the series of patients with adenocarcinoma of the ovary referred to in Fig. 1. For technical and practical reasons, not all cell-maker assays were carried out on each patient or cell fraction. In particular, B-cell assays were infrequent. SIg values were usually difficult to determine as ascites and tumour-derived monocyte–macrophge cells reacted strongly with the $(Fab^1)_2$ reagent, even when there was no reaction with normal blood monocytes.

The results (Fig. 4) indicate several points which are important in establishing the usefulness of our methodology for

FIG. 4.—Immunocytochemical markers associated with blood and blood-equivalent fractions isolated from solid and ascites ovarian tumours. Tumour-derived material was isolated by sedimentation velocity Cells were chosen for values < 6 mm/h. (E) E RFC; (Eest) E RFC stained with ANAE (plotted as % of E RFC); (EA) EA RFC, (NSE) monocytes stained with non-specific esterase reagent and (SIg) B with fluorescein-conjugated anti HuIgG, A, M reagent. Bars represent the mean of each column.

isolating tumour and ascitic inflammatory cells. First, both ascitic and tumour-infiltrating cells (< 6 mm/h) had T-cell values which averaged at least as high as normal or patient blood. Second, the T-cell subset identified on the basis of ANAE staining of E RFC preparations was abnormally low in frequency in the tumour derived material. Third, monocyte levels assessed by nonspecific-esterase staining were usually much lower in the ascitic fraction than in the other groups. Fourth, FcR rosette assays, which detected monocytes only in blood, (Fig. 2, top) were markedly higher in patient material, and apparently measured either esterase-negative monocytes and/or lymphocytes with abnormally high FcR levels, Finally, plasma cells were rarely seen in either ascites or tumour material. In summary, the marker studies indicated that the fractionation procedure yields preparations adequate for comparison with blood-derived cells for tests of immune competance, though the proportions of the various cell types vary greatly between patients. The use of collagenase on all the solid-tumour material and several of the ascites which had gelled is unlikely to have had any effect on these results as we (Haskill *et al.*, 1982; Hayry & Totterman, 1978) have shown this enzyme to be without marked influence on either functional or membrane studies.

*Characterization of infiltration: macrophage fraction*

Cells sedimenting more rapidly than 6 mm/h were pooled on the basis of EA RFC distribution, and a macrophage fraction isolated either by EA RFC separation or adherence. The data summarized in Fig. 5 indicate: first, that highly enriched fractions of macrophages can be isolated from both ascitic and tumour material; second, only in a few instances are the macrophages composed of recently arrived peroxidase-positive monocytes; and third, tumour macrophages are less often peroxidase-positive

FIG. 5.—Immunocytochemical markers associated with macrophage fractions isolated from solid and ascites ovarian tumours. Cells sedimenting > 6 mm/h were further fractionated by adherence or EA RFC removal. EA RFC (EA), myeloperoxidase (MPer), nonspecific esterase (NSE), and acid phosphatase (AP).

than those in the ascites, and show a far greater variation in reactivity for acid phosphatase.

### DISCUSSION

Histological grade, stage, and amount of residual tumour are likely to be the most important prognostic indicators. However, there is considerable evidence, both in animal models and clinical studies, that the degree of mononuclear-cell infiltration may be relevant to survival. Recently there has been an increase in interest in the possible functions of immune cells isolated from solid tumours. Although a variety of effector mechanisms including ADCC, NK, CTL, cytostatic and cytolytic macrophages have been isolated from highly immunogenic animal tumours (Russell *et al.*, 1980; Haskill *et al.*, 1979; Herberman *et al.*, 1980), there are fewer instances where active cells have been isolated from human tumours (Mantovani *et al.*, 1979; Werkmeister *et al.*, 1979; Totterman *et al.*, 1980; Klein *et al.*, 1980; Herberman *et al.*, 1980; and Vose, 1978).

Isolation of a representative sample of inflammatory cells from tumour and ascitic material for functional studies is often difficult. One of the commonest problems is the preselection of effector cell types by cell-fractionation procedures, *e.g.* nylon-wool columns, adherence, and Ficoll–Hypaque density gradients. We have previously demonstrated the value of sedimentation velocity at unit gravity in isolating all the tumour and tumour-infiltrating cells after dispersion in collagenase DNase (Haskill *et al.*, 1979; Becker & Haskill, 1980*b*). Velocity fractions selected for size equivalence to blood-derived cells permit testing for a wide variety of potential effector cell types. In this way, the function of blood-equivalent cells can be determined before identification of the particular cell type or sub-class associated with this activity. Our results are based upon cell fractions which are as representative a mixture as possible of all the inflammatory cells. Thus, apparent differences between our data and others probably result from differences in isolation sequences.

A number of groups have investigated both cytological and functional activity of inflammatory cells in either tumour or ascites-derived material from a variety of human tumours. Several features of these reports warrant discussion, in view of our subsequent reports concerning functional activity associated with these fractions.

Klein *et al.* (1980) have made an extensive study of lymphocyte function in a number of types of human tumours. Using a combination of approaches, in volving velocity, density and adherence, for cell isolation, these authors isolated lymphoid fractions which averaged 51% E RFC and 22% EA RFC cells for their functional studies. They seldom encountered B lymphocytes in these preparations.

Hayry & Totterman (1978) have investigated the types of cell infiltrating several classes of human tumour, including two with ovarian cancer. Infiltrating cells were isolated through a series of steps similar to those described in this report. T cells, monocytes and macrophages, polymorphs and plasma cells were common isolates of these tumours, and there was considerable variation between tumours. The values for the percentage of T cells varied from 35% for several tumour classes to 83% for 3 seminoma patients. The ANAE T-cell subset was the most prominent subset in the isolate.

Recently Mantovani *et al.* (1980*b*) have reported their findings on NK activity associated with malignant effusions of the ovary. Enriched preparations of ascites-associated lymphocytes were obtained by stepwise density and velocity separations similar to those used by Vose *et al.* (1977) followed by repeated adherence steps or discontinuous Ficoll–Hypaque gradients. The percentage of lymphoid cells forming E RFC (39%) averaged less than that in either blood from patients or normal blood. In contrast, the values we have found for E RFC in similar fractions average 78%. The 2-fold difference does not appear to be related to the E RFC assay, as values for normal and patient bloods are similar to ours.

This report demonstrates that mononuclear cell fractions containing >70% T cells are routinely obtained by sedimentation-velocity separation of collagenase-dispersed tumours. Similar separations on ascitic material yielded mostly T cells. Our data however, indicate differences in the T-cell characteristics of these fractions. Several studies have clearly demonstrated that T cells can be further subdivided by a highly localized esterase "dot" staining on E RFC preparations (ANAE positivity) (Saksela *et al.*, 1979*a*; Moretta *et al.*, 1979; Bevan *et al.*, 1980). We have found that the T subset characterized by ANAE reactivity was noticeably smaller in the tumour infiltrate than in patient blood or ascites. Whether this indicates a selective localization or degranulation *in vivo* (Moretta *et al.*, 1979) is not known. This result may, however, provide evidence of a preferential subset localization in ovarian tumours.

The present study also clearly indicates the heterogeneity of the macrophage component of tumour and ascites preparations. Esterase-positive monocyte-sized macrophages were frequently absent from ascites isolates, but were commonly found in tumour-derived material. In contrast, macrophages high in esterase and acid-phosphatase but low in myeloperoxidase activity were isolated from the higher-velocity fractions. These cells varied greatly between patients in their peroxidase and acid-phosphatase activity. In addition, many of the larger ascites macrophages contained phagocytosed leuccytes. Tumour-derived macrophages frequently contained debris, but were seldom peroxidase-positive, and usually had less acid phosphatase. This variation between patients will undoubtedly explain in part the variation in cytolytic activity reported for ascites macrophages derived from ovarian-cancer patients (Mantovani *et al.*, 1980*a*) and serves as an indication that quantitative and qualitative differences in infiltration must be taken into account in assessing intra-tumour immune reactivity.

In the accompanying reports, the NK, ADCC, PHA and suppressor-cell responses of the mononuclear cell fractions described above will be outlined. These data indicate the frequent dissociation between systemic and *in situ* immunity, as well as the decreased reactivity of some classes of tumour-infiltrating cells isolated from ovarian cancers.

This work was supported by the United States Public Health Service Grant CA-23648 to S.H. and by Gynecologic Oncology Groups Project Grant 2-R10-CA 23073-03 to W. F. and L. W.

REFERENCES

Becker, S. & Haskill, J. S. (1980*a*) Non T-cell mediated cytotoxicity in MSV tumor-bearing mice. III. Macrophage-mediated cytotoxicity against autochthonous MSV tumor-isolated target cells. *Int. J. Cancer*, **25**, 535.

Becker, S. & Haskill, J. S. (1980*b*) Characterization of the presumptive sarcoma cells in primary MSV tumors. *Int. J. Cancer*, **25**, 543.

Bevan, A., Burns, G. F., Gray, L. & Cawley, J. C. (1980) Cytochemistry of human T-cell subpopulations. *Scand. J. Immunol.*, **11**, 223.

BOYUM, A. (1968) Separation of lymphocytes and bone marrow. *Scand. J. Clin. Lab. Invest.*, **97**, 77.

BURSTONE, M. S. (1959) Histochemical demonstration of acid phosphatase activity in osteoclasts. *J. Histochem. Cytochem.*, **7**, 39.

HASKILL, J. S. (1981*a*) Collection of macrophages from tumors. In *Manual of Macrophage Methodology*. New York: Dekker. p. 43.

HASKILL, J. S. (1981*b*) Unit gravity sedimentation of tumor-associated macrophages. In *Manual of Macrophage Methodology*. New York: Dekker. p. 81.

HASKILL, J. S., KEY, M., RADOV, L. A. & 7 others (1979) The importance of antibody and macrophages in spontaneous and drug-induced regression of the T1699 mammary adenocarcinoma. *J. Reticuloend. Soc.*, **26**, 417.

HASKILL, J. S., KOREN, H., BECKER, S., FOWLER, W. & WALTON, L. (1982) Mononuclear cell infiltration in ovarian cancer. II. *Br. J. Cancer*, **45**, 737.

HAYRY, P. & TOTTERMAN, T. H. (1978) Cytological and functional analysis of inflammatory infiltrates in human malignant cells. I. Composition of the inflammatory infiltrates. *Eur. J. Immunol.*, **8**, 866.

HERBERMAN, R. B., HOLDEN, H. I., VARESIO, L. & 7 others (1980) Immunologic reactivity of lymphoid cells in tumors. In *Contemporary Topics in Immunobiology*, Vol. 10 (Eds. Witz & Hanna). New York: Plenum Press. p. 61.

HOFFMAN, T. & KUNKEL, H. G. (1976) The E-rosette test in *in vitro* methods. In *Cell Mediated and Tumor Immunity* (Eds. Bloom & David). New York: Academic Press. p. 71.

IOACHIM, H. L. (1976) The stromal reaction of tumors: An expression of immune surveillance. *J. Natl Cancer Inst.*, **57**, 465.

KAPLOW, L. S. (1965) Simplified myeloperoxidase stain using benzidine dihydrochloride. *Blood*, **26**, 215.

KLEIN, E., VANKY, F., GALILI, U., VOSV, B. M. & FOPP, M. (1980) Separation and characteristics of tumor-infiltrating lymphocytes in man. In *Contemporary Topics in Immunobiology*, Vol. 10 (Eds. Witz & Hanna). New York: Plenum Press. p. 79.

KORN, J. H., HASKILL, J. S., HOLDEN, H. T., RADOV, L. A. & RITTER, F. L. (1978) *In situ* Fc receptor-bearing cells in two murine tumors. I. Isolation and identification. *J. Natl Cancer Inst.*, **60**, 1390.

MANTOVANI, A., GIUSEPPE, P., POLENTARUTTI, N., BOLIS, G., MANGIONI, C. & SPREAFICO, F. (1979) Effects on *in vitro* tumor growth of macrophages isolated from human ascitic ovarian tumors. *Int. J. Cancer.*, **23**, 157.

MANTOVANI, A., POLENTARUTTI, N., PERI, G. & 4 others (1980*a*) Cytotoxic activity on tumor cells of peripheral blood monocytes and tumor-associated macrophages in patients with ascitic ovarian tumors. *J. Natl Cancer Inst.*, **64**, 1307.

MANTOVANI, A., ALLAVENA, P., SESSA, C., BOLIS, G. & MANGIONI, C. (1980*b*) Natural killer activity of lymphoid cells isolated from human ascitic ovarian tumors. *Int. J. Cancer.*, **25**, 573.

MILLER, R. G. & PHILLIPS, R. A. (1969) Saparation of cells by velocity and sedimentation. *J. Cell Physiol.*, **73**, 191.

MORETTA, L., MINGARI, M. C., MORETTA, A. & COOPER, M. D. (1979) Human T lymphocyte subpopulation studies of the mechanism by which T cells bearing Fc receptors for IgG suppress T-dependent B cell differentiation induced by pokeweed mitogen. *J. Immunol.*, **122**, 984.

NEWMAN, C. E., FORD, C. H. J. & JORDAN, J. A. (Eds) (1980) *Ovarian Cancer Advances in the Biosciences*, Vol. 26. New York: Pergamon Press.

RUSSELL, S. W., GILLESPIE, G. Y. & PACE, J. L. (1980) Evidence for mononuclear phagocytes in solid neoplasms and appraisal of their nonspecific cytotoxic capabilities. In *Contemporary Topics in Immunobiology*, Vol. 10 (Eds. Witz & Hanna). New York: Plenum Press. p. 79.

SAKSELA, E., TIMONEN, T., RANKI, A. & HAYRY, P. (1979*a*) Morphological and functional characterization of isolated effector cells responsible for human natural killer activity to fetal fibroblasts and to cultured cell line targets. *Immunol. Rev.*, **44**, 71.

TOTTERMAN, T. H., PARTHENAIS, E., HAYRY, P., TIMONEN, T. & SAKSELA, E. (1980) Cytological and functional analysis of inflammatory infiltrates in human malignant tumors. III. Further functional investigations using cultured autochthonous tumor cell lines and freeze-thawed infiltrating inflammatory cells. *Cell. Immunol.*, **54**, 219.

UNDERWOOD, J. C. E. (1974) Lymphoreticular infiltration in human tumours: Prognostic and biological implications: A review. *Br. J. Cancer*, **30**, 538.

VOSE, B. M., VANKY, F., ARGOV, S. & KLEIN, E. (1977) Natural cytotoxicity in man: Activity of lymph node and tumor-infiltrating lymphocytes. *Eur. J. Immunol.*, **7**, 753.

VOSE, B. M. (1978) Cytotoxicity of adherent cells associated with some human tumours and lung tissues. *Cancer Immunol. Immunother.*, **5**, 173.

WERKMEISTER, J. A., PIHL, E., NIND, A. P., FLANNERY, G. R. & NAIRN, R. C. (1979) Immunoreactivity by intrinsic lymphoid cells in colorectal carcinoma. *Br. J. Cancer*, **40**, 839.